# Multimodal evidence for large-scale network imbalance in etomidate use disorder

**DOI:** 10.1093/ijnp/pyag049

**Published:** 2026-10-14

**Authors:** Juan Le, Xingmin Wang, Ying Tang, Hao Chen, Xinxin Chen, Xuhao Wang, Yunde Tang, Lishun Zhao, Lin Zhao, Hongxian Shen, Qiuping Huang, Zhenjiang Liao

**Affiliations:** Department of Psychiatry, National Clinical Research Center for Mental Disorders, The Second Xiangya Hospital of Central South University, Changsha, Hunan, China; Department of Psychiatry, National Clinical Research Center for Mental Disorders, The Second Xiangya Hospital of Central South University, Changsha, Hunan, China; Department of Psychiatry, National Clinical Research Center for Mental Disorders, The Second Xiangya Hospital of Central South University, Changsha, Hunan, China; Department of Psychiatry, National Clinical Research Center for Mental Disorders, The Second Xiangya Hospital of Central South University, Changsha, Hunan, China; Department of Psychiatry, The Second People’s Hospital of Hunan Province (Brain Hospital of Hunan Province), Changsha, Hunan, China; Department of Psychiatry, National Clinical Research Center for Mental Disorders, The Second Xiangya Hospital of Central South University, Changsha, Hunan, China; Hunan Lituo Compulsory Isolation and Drug Rehabilitation Center, Hunan Lituo Drug Rehabilitation and Recovery Center, Changsha, Hunan, China; Department of Psychiatry, National Clinical Research Center for Mental Disorders, The Second Xiangya Hospital of Central South University, Changsha, Hunan, China; Department of Psychiatry, Hunan Provincial Lushan Compulsory Isolation Detoxification Center, Changsha, Hunan, China; Department of Psychiatry, National Clinical Research Center for Mental Disorders, The Second Xiangya Hospital of Central South University, Changsha, Hunan, China; Department of Psychology, Hunan University of Chinese Medicine, Changsha, Hunan, China; Department of Psychiatry, National Clinical Research Center for Mental Disorders, The Second Xiangya Hospital of Central South University, Changsha, Hunan, China

**Keywords:** etomidate use disorder, electroencephalography, functional near-infrared spectroscopy, functional connectivity, machine learning

## Abstract

**Background:**

Etomidate use disorder (EUD) is an escalating public health issue in East and Southeast Asia, yet its neuroimaging features remain poorly understood. This study used simultaneous electroencephalography (EEG) and functional near-infrared spectroscopy (fNIRS) to examine multimodal functional connectivity alterations in individuals with EUD.

**Methods:**

A total of 54 individuals with EUD and 46 healthy controls (HCs) were recruited and underwent simultaneous resting-state EEG and fNIRS recordings. Psychological dimensions including impulsivity, aggression, and craving were evaluated using standardized and validated scales. Source-level EEG functional connectivity and fNIRS hemodynamic connectivity were analyzed to identify group differences. Associations between altered connectivity features and clinical measures were examined within the EUD group. Machine learning analyses were conducted as exploratory assessments of the discriminative information contained in multimodal connectivity features.

**Results:**

In unadjusted exploratory analyses of Barratt Impulsiveness Scale, 11th Edition and Buss-Perry Aggression Questionnaire subscales, the EUD group showed higher cognitive impulsivity, non-planning impulsivity, physical aggression, anger, and self-directed aggression (*t* = 2.32-4.54, *P* < .05), but lower motor impulsivity, compared with HCs. Source-level EEG analysis revealed significant group differences only in the delta band, with reduced large-scale connectivity in EUD involving default mode, dorsal attention, visual and sensorimotor systems. Functional near-infrared spectroscopy analysis showed reduced hemodynamic connectivity across multiple frontal subregions and motor-related areas. Weaker connectivity between the primary motor cortex and the triangular part of the inferior frontal gyrus was associated with stronger negative reinforcement craving (rho = −0.471, *p_*FDR < 0.05). Exploratory machine learning analysis showed preliminary discriminative performance, with the random forest model producing the numerically highest test-set area under the curve (AUC, 0.80) in this particular data split.

**Conclusion:**

Convergent EEG-fNIRS findings suggest delta-band network decoupling and reduced frontal hemodynamic connectivity as multimodal neural features of EUD. These findings provide preliminary evidence for the neural mechanisms of EUD and may inform future objective assessment and treatment-monitoring studies.

## Graphical Abstract

**Graphical Abstract:**
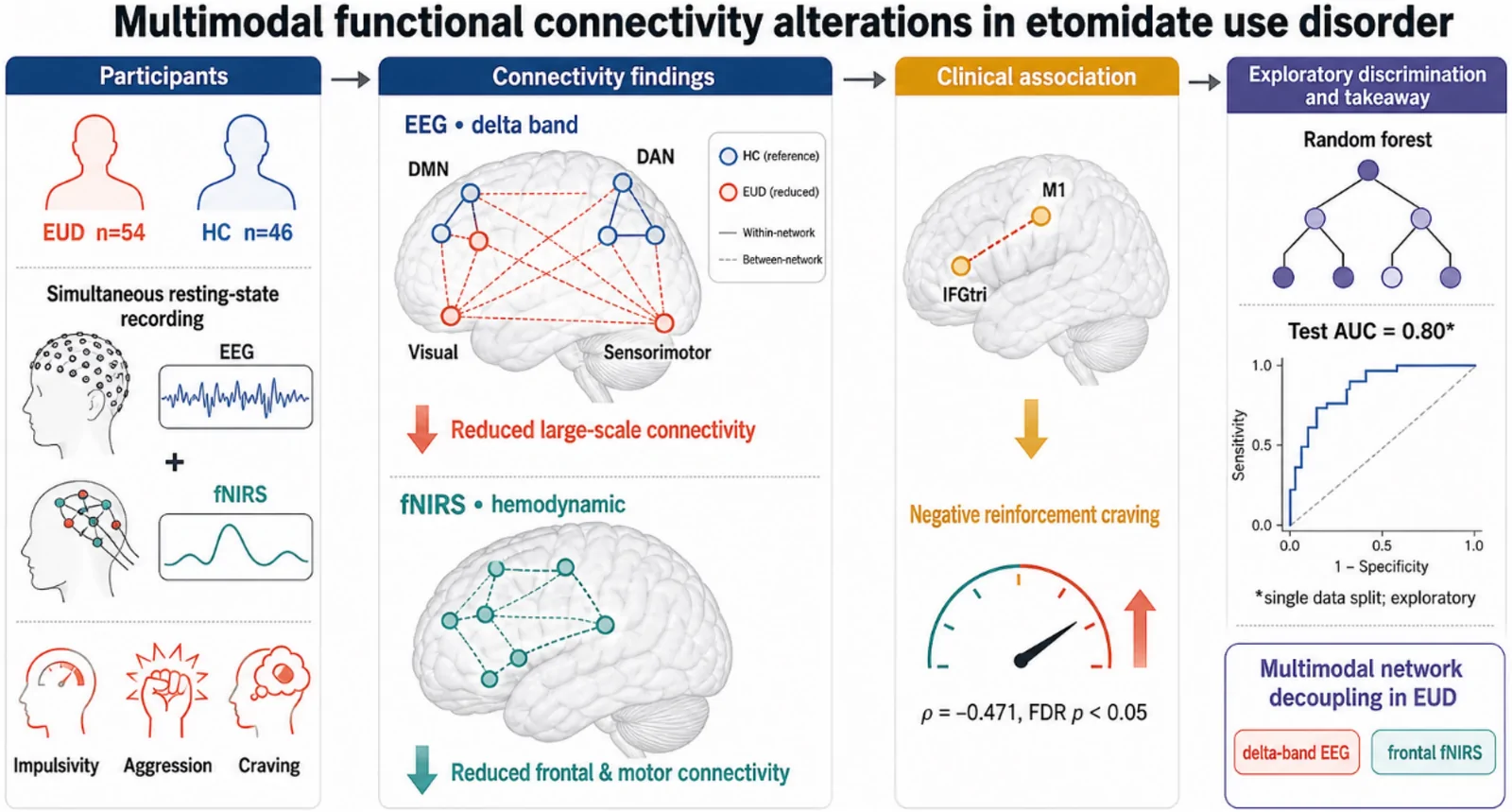

## Teaser

**Highlights**
 

- Simultaneous electroencephalography-functional near-infrared spectroscopy revealed impaired functional integration in etomidate use disorder (EUD).
- Etomidate use disorder was associated with delta-band decoupling across large-scale networks.
- Weakened prefrontal-motor connectivity may be associated with negative reinforcement craving.

**Significant outcomes**

- This study provides convergent electroencephalography-functional near-infrared spectroscopy evidence that etomidate use disorder (EUD) is associated with impaired functional integration, including decoupling of the default-mode network and the dorsal attention network in the delta band, and reduced frontal hemodynamic connectivity.
- Weakened prefrontal-motor connectivity may be associated with stronger negative reinforcement craving, linking multimodal network alterations to a clinically relevant addiction phenotype.
- Exploratory machine learning analyses suggested that multimodal connectivity features contain discriminative information for EUD, but these findings require validation in independent cohorts.

**Limitations**

- Several limitations should be considered. First, this study recruited only male participants from a single center with a limited sample size, restricting the generalizability of the results. Future multicenter investigations incorporating female individuals and larger cohorts are required to validate our observations.
- Second, all individuals with etomidate use disorder (EUD) were assessed during early withdrawal, 3-14 days after their last etomidate use. Therefore, the observed neuropsychological and connectivity abnormalities may reflect a combination of trait vulnerability, repeated etomidate exposure and withdrawal-related state effects. The cross-sectional design prevents these possibilities from being distinguished.
- Third, although simultaneous electroencephalography-functional near-infrared spectroscopy provides complementary electrophysiological and hemodynamic information, both modalities have limited sensitivity to subcortical structures that are central to addiction, including the striatum and amygdala. Future studies integrating EEG-fNIRS with structural and functional Magnetic resonance imaging (MRI) may provide a more complete account of cortico-subcortical circuitry in EUD.
- Fourth, residual confounding may arise from unadjusted clinical variables (etomidate use duration, daily dose, comorbid anxiety/depression, nicotine exposure) and the absence of objective wakefulness monitoring during eyes-closed recordings.
- Finally, the machine learning analysis was exploratory and lacked external validation. Because the 5 models were compared on a single small held-out test set without formal statistical testing of AUC differences, and because the final model was selected according to the highest cross-validated accuracy across 1000 random seeds, the reported test-set performance may carry selection optimism and should be interpreted cautiously. Larger independent cohorts and longitudinal designs are needed before these measures can be considered clinically useful biomarkers.

## Introduction

Etomidate is an ultrashort-acting, imidazole-derived intravenous nonbarbiturate sedative-hypnotic, widely used for anesthesia induction and procedural sedation due to its rapid onset and relatively stable cardiovascular profile.^1^ Its anesthetic effects are primarily mediated via positive allosteric modulation of γ-aminobutyric acid type A (GABA_A) receptors, producing potent central nervous system inhibition.^2^ Although etomidate has traditionally been considered within the context of anesthesia, its non-medical use has recently emerged as a public health concern in East and Southeast Asia.^1^^,^^3^ In China, more than 33 000 etomidate-related cases were documented in 2024, indicating a rapidly expanding pattern of misuse.^4^ Chronic etomidate exposure has been associated with acute neurological manifestations, endocrine disturbances (adrenal suppression)^5^ and psychiatric comorbidities including mood disorders and cognitive impairment.^6–9^ Despite growing clinical and epidemiological concerns, the neuroimaging mechanisms underlying etomidate use disorder (EUD) remain poorly characterized.

Substance use disorders (SUDs) are chronic, relapsing brain disorders characterized by persistent impairments in behavioral regulation and reward processing. Extensive research indicates that SUDs are closely associated with abnormalities in impulsivity and aggression, as well as psychological craving.^10–12^ Impulsivity, a core trans-diagnostic feature of addiction, reflects impaired top-down inhibitory control within prefrontal–striatal circuits, whereby weakened prefrontal regulation enhances sensitivity to drug-related cues and promotes compulsive drug-seeking behavior.^13^^,^^14^ Similarly, aggression-particularly anger expression and self-directed aggression—is significantly more prevalent in individuals with SUDs than in healthy controls (HCs),^15^ and has been linked to dysfunctional connectivity among prefrontal, parietal, and limbic regions.^16^ Craving further extends this framework because it is not merely a subjective desire for drug use, but a network-level state involving dysregulated interactions among prefrontal control regions, salience-related systems, and mesolimbic reward circuitry.^17^ Collectively, these behavioral phenotypes converge on large-scale brain systems governing cognitive control, salience attribution, and reward valuation, suggesting that addiction may be conceptualized as a disorder of distributed network imbalance rather than isolated regional abnormalities.

Because impulsivity, aggression, and craving are expressed through distributed neural systems, neuroimaging provides a necessary bridge between clinical phenotypes and circuit-level mechanisms in SUDs.^18^^,^^19^ Resting-state fMRI studies have implicated corticostriatal-thalamocortical circuits in addiction,^20^ whereas EEG studies have identified electrophysiological markers associated with abstinence, craving and treatment outcomes.^21^

While fMRI and EEG studies have yielded valuable insights into various SUDs, neuroimaging research specifically targeting EUD remains very limited. Notably, recent EEG-fNIRS work has shown that multimodal signal fusion can distinguish individuals with EUD from HCs, providing an important methodological precedent.^22^ A multimodal approach is particularly relevant for EUD because chronic exposure to a GABAergic anesthetic agent may affect both electrophysiological synchrony and hemodynamic coupling. Eelectroencephalography provides millisecond-level sensitivity to oscillatory activity and frequency-specific functional connectivity (FC), but its spatial localization is limited by volume conduction.^23^ Functional near-infrared spectroscopy complements EEG by measuring cortical hemodynamic responses with relatively greater spatial specificity in superficial cortical regions, particularly the prefrontal cortex.^24^ Simultaneous EEG-fNIRS recording therefore allows electrophysiological and hemodynamic abnormalities to be examined within the same resting-state condition. However, systematic EEG-fNIRS studies linking multimodal connectivity abnormalities to clinical phenotypes in EUD are still lacking.

Machine learning (ML) approaches have increasingly been applied in psychiatric research to integrate high-dimensional neuroimaging and behavioral data, facilitating the identification of disorder-related network patterns and potential biomarkers in major depressive disorder, schizophrenia, and methamphetamine use disorder.^25–27^ Despite these advances, no studies to date have systematically applied ML to multimodal EEG-fNIRS connectivity features to characterize EUD, nor has frequency-specific network information been comprehensively evaluated. Moreover, existing research on etomidate has largely focused on its anesthetic properties, with limited attention paid to its addictive potential and associated neural circuitry.^28^^,^^29^

Building on this rationale, the present study used simultaneous resting-state EEG and fNIRS to examine multimodal FC abnormalities in individuals with EUD. We focused on 3 linked questions. First, we tested whether EUD is associated with frequency-specific large-scale network alterations in source-level EEG connectivity. Second, we examined whether fNIRS reveals abnormal hemodynamic coupling within prefrontal circuits involved in inhibitory control, reward valuation, and motor preparation. Third, we assessed whether these multimodal connectivity features are associated with core clinical phenotypes, including impulsivity, aggression, and craving. As an exploratory translational analysis, we further evaluated whether EEG-fNIRS connectivity features could support individual-level classification of EUD using machine learning. We hypothesized that EUD would be characterized by impaired brain network integration, and that these alterations would relate to craving and behavioral dysregulation.

## Methods

### Participants

This study recruited individuals with EUD from a rehabilitation center in Hunan Province between June and December 2023 (where patients are admitted via compulsory judicial referral or voluntary admission). During the study period, the center admitted only male patients; therefore, only male participants were eligible for enrollment. Study participations were entirely voluntary. Refusal to participate or withdrawal from the study did not affect clinical care, legal status, judicial procedures or access to services.

Inclusion criteria for the EUD group were as follows: (1) meeting the Diagnostic and Statistical Manual of Mental Disorders, Fifth Edition (DSM-5) criteria for SUDs (etomidate),^30^ the early withdrawal period, 3-14 days following their last etomidate use; (2) using etomidate at least twice per month for the past year, continuously for 3 or more months with a positive urine test; (3) aged 16 to 40 years, male, and right-handed. Exclusion criteria included: (1) poly-substance abuse (use of psychoactive substances or narcotics within the past year); (2) participation in other addiction-related intervention programs; (3) presence of severe physical illness requiring regular medication; and (4) positive screening for major psychiatric disorders using the Mini International Neuropsychiatric Interview.^31^^,^^32^

Healthy controls (HCs) were recruited from the local community via targeted online advertisements. Inclusion criteria for the HCs group were as follows: (1) no serious somatic or neurological diseases; (2) no history of drug use; and (3) not meeting the diagnostic criteria for any mental illness in the DSM-5.^30^ All assessments were conducted by 2 board-certified psychiatrists trained in addiction medicine.

Participants received RMB 200 as reimbursement for their time costs. This compensation scheme was approved by the institutional ethics committee, and was provided regardless of study completion and not contingent on any clinical, behavioral or neuroimaging outcome. Written informed consent was obtained from all adult participants. Seven participants aged 16-17 years (7% of the total sample) were enrolled; for these minors, written informed consent was obtained from their legal guardians, and written assent was obtained from each participant. The study protocol, including recruitment from compulsory rehabilitation settings, was approved by the Ethics Committee of the Second Xiangya Hospital, Central South University (No. 2023K003) and conducted in accordance with the Declaration of Helsinki.

Of the 54 EUD subjects included in this study, 46 were from the same cohort reported by Gao et al.,^22^ and their original synchronized EEG-fNIRS data were reused for this analysis. However, an independent analytical framework was employed, and the HCs groups in the 2 studies were completely distinct. The study by Gao et al.^22^ focused on methodological neurovascular coupling fusion analysis, whereas this study focused on large-scale network imbalance and clinical relevance analysis.

### Neuropsychological assessment

#### Clinical and substance-use assessment

Qualified psychiatrists conducted a clinical evaluation for EUD using DSM-5 criteria. The assessment included 11 binary-scored items (1 = positive, 0 = negative); a diagnosis required at least two symptoms within the past 12 months. Demographic information on age and educational attainment was recorded.

#### Impulsivity

The Chinese version of the Barratt Impulsiveness Scale, 11th Edition (BIS-11) was used to assess participants’ impulsive traits, measuring 3 dimensions: cognitive, motor, and non-planning impulsivity. A 4-point Likert scale was used (1 = Almost Never, 4 = Almost Always), with higher scores indicating more prominent impulsive traits. The scale has shown good reliability and validity in substance-dependent populations, with a Cronbach’s α coefficient of 0.79-0.83.^33^

#### Aggression

Aggression was assessed using the Chinese version of the Buss-Perry Aggression Questionnaire (BPAQ). The BPAQ contains 30 items and evaluates 5 aggression-related dimensions: physical aggression, verbal aggression, anger, hostility, and self-directed aggression. Items are rated on a 5-point Likert scale, with higher scores indicating greater levels of aggression (Cronbach’s α = 0.60-0.89).^34^

#### Craving

Craving was assessed using the Desire for Drug Questionnaire (DDQ). The DDQ contains 13 items rated on a 7-point Likert scale and evaluates 3 dimensions of craving: drug craving and intention, negative reinforcement, and drug control. The scale has demonstrated good reliability and validity in studies involving various addictive substances across multiple countries (Cronbach’s α = 0.86).^35^

### Data acquisition

During the experiment, participants were seated comfortably in a quiet, temperature-controlled laboratory, with instructions to close their eyes, relax, and remain awake. Simultaneous 5-min recordings of EEG and fNIRS signals were acquired. EEG data were recorded using a 64-channel wireless system (NeuSen W, Neuracle, China), with electrode placement following the international 10-10 system and a sampling rate of 1 kHz. Electrode impedances were kept below 10 kΩ.

Functional near-infrared spectroscopy signals were collected using a 51-channel system (NirSmart-6000A, Danyang Huichuang Medical Equipment Co., Ltd., China) with 23 emitters and 16 detectors. Dual-wavelength lasers (730 and 850 nm) detected concentration changes in oxyhemoglobin and deoxyhemoglobin at an 11 Hz sampling rate. Channel coordinates were assigned based on the international 10-20 EEG system, then transformed into Montreal Neurological Institute (MNI) space using the Nirspace algorithm (Danyang Huichuang Medical Equipment Co., Ltd., Jiangsu, China). This system enabled accurate anatomical localization by projecting channel positions onto the MNI standard brain template. The placement of the EEG-fNIRS detection cap used in this study is shown in Figure 1.

**Figure 1:**
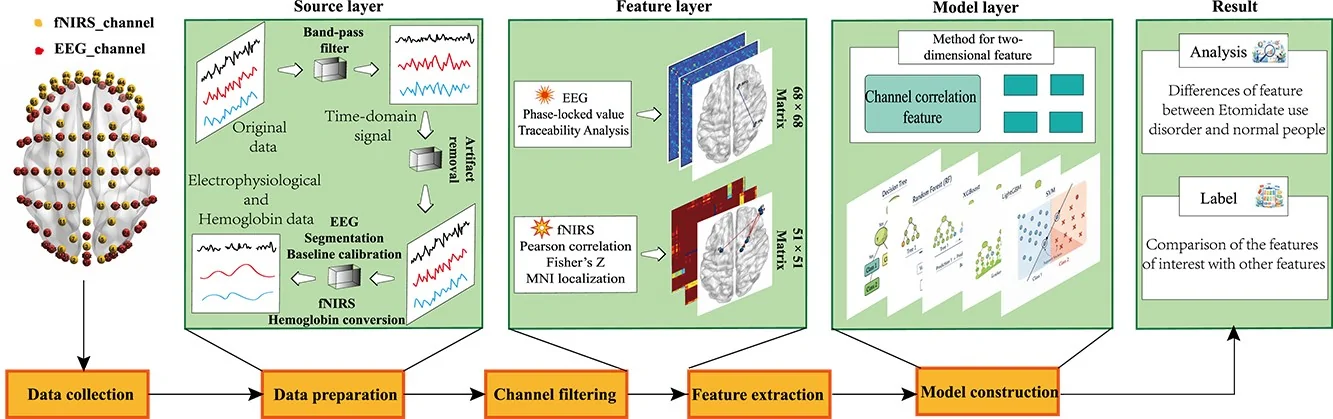
Flowchart of the EEG-fNIRS data processing and analysis. EEG-fNIRS, electroencephalography-functional near-infrared spectroscopy.

### Data preprocessing

EEG preprocessing was performed using EEGLAB 2023b^36^ in MATLAB 2023b. Raw EEG data were downsampled to 250 Hz. Five disconnected ECG/EOG channels were removed, leaving 59 EEG channels for further analysis. The data were then band-pass filtered at 1-45 Hz and notch filtered at 48-52 Hz to reduce line noise. Continuous EEG recordings were segmented into 2-s epochs. Bad channels were identified and interpolated. Independent component analysis was then applied, and components reflecting ocular or myogenic artifacts were removed by visual inspection. Epochs with amplitudes exceeding ±100 μV were automatically rejected. The remaining data were re-referenced to the whole-brain average reference.

Raw fNIRS data were preprocessed using the Homer2 toolbox in MATLAB 2023b.^37^ Motion artifacts were removed using a modified spline method with the following parameters: STDEVthresh = 50, AMPthresh = 5, TMotion = 2 s, and TMASK = 4 s.^38^ Specifically, a sliding window was employed to calculate the local variance of fNIRS signals, and segments exceeding the threshold were classified as motion artifacts. Spline interpolation was then applied to these segments to correct baseline drift. Residual transient spikes were detected and corrected to minimize their influence on subsequent FC analyses. A 0.01-0.10 Hz bandpass filter was applied to remove physiological noise.^39^ The optical signals were then converted into hemodynamic parameters (ie, oxyhemoglobin and deoxyhemoglobin concentration changes) using a modified Beer–Lambert law.^40^^,^^41^ Since Δ[HbO2] provides a higher signal-to-noise ratio compared to Δ[Hb], only HbO2 data were analyzed in this study.

### E‌EG source reconstruction

Post-preprocessing of EEG data was performed using the BrainStorm toolbox within MATLAB 2023b.^42^^,^^43^ A forward head model was computed using OpenMEEG. Cortical source activity was then estimated using the sLORETA algorithm implemented in Brainstorm. The source space projections for each subject were then projected (registered) onto the MNI/Colin27 template brain surface for standardization. The 15 000-voxel source space was segmented into 68 cortical regions of interest (ROIs) based on the Desikan–Killiany neuroanatomical atlas.^44^ For each participant, regional source time series were obtained by averaging the reconstructed cortical activity within each ROI. These ROI-level time series were used for subsequent frequency-specific EEG FC analyses.

### Feature extraction and machine learning

#### E‌EG functional connectivity

The averaged temporal source activity within each ROI was bandpass-filtered into 5 frequency bands: delta (1-4 Hz), theta (4-8 Hz), alpha (8-12 Hz), beta (13-30 Hz), and low gamma (30-45 Hz). For each frequency band, the Hilbert transform was applied to calculate instantaneous phase time series. Functional connectivity between all ROI pairs was estimated via phase-locking value, resulting in a 68 × 68 connectivity matrix for each frequency band. Network-based statistic (NBS) was adopted to detect significant group differences in connectivity. The edge-level threshold was set at *P* = .001, and connected network components were considered statistically significant at component-level threshold *P* = .05, based on 5000 permutations.

#### fNIRS functional connectivity

For the fNIRS signals, we first calculated the correlation coefficient matrix between the time series of the 51 channels using Pearson correlation analysis. Subsequently, Fisher’s Z-transformation was applied to this matrix for nonlinear correction to ensure a normal distribution, thereby obtaining a FC matrix for subsequent analysis. Between-group differences in fNIRS FC were assessed using the NBS approach with the same permutation parameters described for the EEG connectivity analysis. Significant channel-level connections were anatomically labeled according to their corresponding Brodmann areas based on the registered channel coordinates. The resulting EEG-fNIRS connectivity networks were visualized using BrainNet Viewer 2018 (http://www.nitrc.org/projects/bnv/). The overall EEG-fNIRS processing and analysis workflow is shown in Figure 1.

#### Machine learning

ML analyses were conducted as an exploratory translational analysis to evaluate whether multimodal FC features could discriminate individuals with EUD from HCs. All analyses were implemented in Python 3.9 using scikit-learn v1.6.1, together with the XGBoost and LightGBM packages. Participants were randomly divided into training and independent test sets at a 7:3 ratio. The independent test set was held out during feature selection, preprocessing and hyperparameter tuning, and was used only for final performance evaluation. Functional connectivity values were used as candidate features. Within the training set only, independent two-sample *t*-tests were applied to rank connectivity features according to the absolute *t* statistic, and the top 20 features were retained for model training. This univariate procedure was used only for feature ranking and was not interpreted as inferential statistical testing. For algorithms requiring feature scaling, including support vector machine (SVM), Z-score standardization parameters were estimated from the training set and then applied to the test set. Five classification algorithms were evaluated: decision tree, random forest, XGBoost, LightGBM, and SVM. Hyperparameters were optimized within the training set using grid search with 10-fold cross-validation, with mean accuracy as the optimization criterion. To mitigate the influence of random seed initialization on model performance, the grid search procedure was repeated 1000 times with different random seeds. The model with the highest cross-validation accuracy among these 1000 repetitions was then refitted on the full training set and evaluated once on the independent test set. Model performance was quantified using accuracy, precision, recall, F1 score, and area under the receiver operating characteristic curve (AUC). The 95% confidence interval for AUC was estimated via bootstrap resampling of the test set predictions (1000 resamples). Confusion matrices and receiver operating characteristic (ROC) curves were generated to visualize classification performance.

### Statistical analyses

Statistical analyses were performed using IBM SPSS Statistics 27.0. Continuous variables were tested for normality using the Shapiro–Wilk test. Normally distributed data are presented as mean ± SD. Between-group comparisons were conducted using independent samples *t*-tests or Mann–Whitney U tests. Accordingly, between-group differences in age and educational attainment were first tested via independent samples *t*-tests. These variables were included as covariates in subsequent analyses only if statistically significant. Group differences in EEG and fNIRS FC were evaluated via the NBS with permutation testing to correct for multiple comparisons across edges. Within the EUD group, Spearman rank correlations were computed between NBS-significant connections and neuropsychological measures, with False discovery rate (FDR) correction applied within 4 a priori families stratified by imaging modality (EEG delta/fNIRS) and clinical construct (substance-related/behavioral scales). All tests were two-tailed, with statistical significance set at *P* < .05; uncorrected associations are reported as exploratory findings and interpreted cautiously.

## Results

### Demographics and clinical measures

A total of 100 male participants were included in the final analysis, comprising 54 patients with EUD and 46 HCs. The median withdrawal period of participants in the EUD group was 9.00 (7.00-12.00) days. No statistically significant between-group difference was detected in age and years of education (*t* = −1.64/*t* = −1.84, *P* > .05). For impulsivity, the BIS-11 total score did not differ significantly between the EUD and HCs (*t* = 1.96, *P* > .05). For subdimensions, the EUD group scored significantly higher than the HCs on cognitive impulsivity (*t* = 3.25, *P* < .01) and non-planning impulsivity (*t* = 2.32, *P* < .05). Conversely, the HCs had a significantly higher motor impulsivity score than the EUD group (*t* = −2.39, *P* < .05). For aggression, the BPAQ score of the EUD group was significantly higher than that of the HCs (*t* = 3.37, *P* < .01). Specifically, the EUD group exhibited significantly higher scores on physical aggression (*t* = 4.54, *P* < .001), anger (*t* = 3.66, *P* < .001), and self-directed aggression (*t* = 3.34, *P* < .01). No significant between-group differences were observed in verbal aggression (*t* = −0.34, *P* > .05) or hostility (*t* = 1.20, *P* > .05) (See Table 1). Because these behavioral comparisons were exploratory, the observed group differences should be interpreted cautiously.

**Table 1:** Demographic information and neuropsychological profiles.

	**EUD (*n* = 54)**	**HCs (*n* = 46)**	***t*/*z***	***P***	**Cohen’s d**
Age (Y)	23.87 ± 5.56	25.46 ± 4.08	−1.64	ns	
Years of education (Y)	11.91 ± 1.93	12.63 ± 1.99	−1.84	ns	
**DDQ**					
Drug craving	2.42 (1.67, 3.17)				
Negative reinforcement	3.20 (2.20, 4.00)				
Drug control	4.00 (3.50, 5.00)				
**Withdrawal period (days)**	9.00 (7.00, 12.00)				
**BIS-11**	94.06 ± 11.38	89.74 ± 10.52	1.96	ns	0.34
Motor impulsivity	21.65 ± 7.06	25.02 ± 7.04	−2.39	^*^	−0.48
Cognitive impulsivity	35.87 ± 5.15	32.50 ± 5.18	3.25	^**^	0.65
Non-planning impulsivity	34.69 ± 7.98	31.22 ± 6.79	2.32	^*^	0.47
**BPAQ**	69.52 ± 17.58	58.30 ± 15.39	3.37	^**^	0.68
Physical aggression	19.41 ± 5.78	14.43 ± 5.05	4.54	^***^	0.91
Verbal aggression	11.93 ± 3.16	12.15 ± 3.52	−0.34	ns	−0.07
Anger	13.61 ± 4.75	10.41 ± 3.85	3.66	^***^	0.73
Hostility	14.72 ± 4.56	13.63 ± 4.58	1.20	ns	0.24
Self-directed aggression	9.85 ± 3.40	7.67 ± 2.99	3.34	^**^	0.68

Continuous data are presented as the mean ± SD or median (range), depending on the distribution of the data. Categorical data are presented as count (percentage).

Abbreviations: BIS-11, Barratt Impulsiveness Scale, version 11; BPAQ, Buss-Perry Aggression Questionnaire; DDQ, Desire for Drug Questionnaire; EUD, etomidate use disorder group; HC, healthy control group. ^*^P < .05, ^**^P < .01, ^***^P < .001, ns: No significant difference. Cohen’s d interpretation criteria: |d| < 0.2-very small; 0.2-0.5 small; 0.5-0.8 moderate; ≥0.8 large; a positive value indicates that EUD is higher, while a negative value indicates that HC is higher. All 10 BIS-11/BPAQ subscale comparisons in this table are unadjusted exploratory analyses. No correction for multiple testing was applied, and these findings should be interpreted cautiously.

### E‌EG functional connectivity

Source-level EEG FC analysis revealed significant group differences only in the delta band (1-4 Hz) after NBS correction, with a component-level NBS-corrected *P* value of .00159968. Suprathreshold components were detected in the theta, alpha, beta, and low-gamma bands at the edge-level threshold, but none survived component-level NBS correction (all component-level corrected *P* values >.05). In the delta band, individuals with EUD showed a significant abnormal subnetwork comprising 21 connections (*t* = −4.68 to −3.45, NBS_corrected *P* = .00159968, see Supplementary Table S1). All significant connections showed lower FC in the EUD group than in the HCs group. The abnormal subnetwork was dominated by cross-network decoupling, which accounted for 85.7% of the altered connections. Eight connections showed relatively strong effects (|*t*| >4; see Figure 2A). The affected core systems included the default mode network (inferior parietal lobule, middle temporal gyrus) and the dorsal attention network (superior parietal lobule); among these, the right inferior parietal lobule and the left superior parietal lobule were the hubs with the strongest effects, and the connections between them, as well as their respective connections with the visual network (cuneus) and the sensorimotor network (transverse temporal gyrus), were all significantly lower in the EUD group.

**Figure 2:**
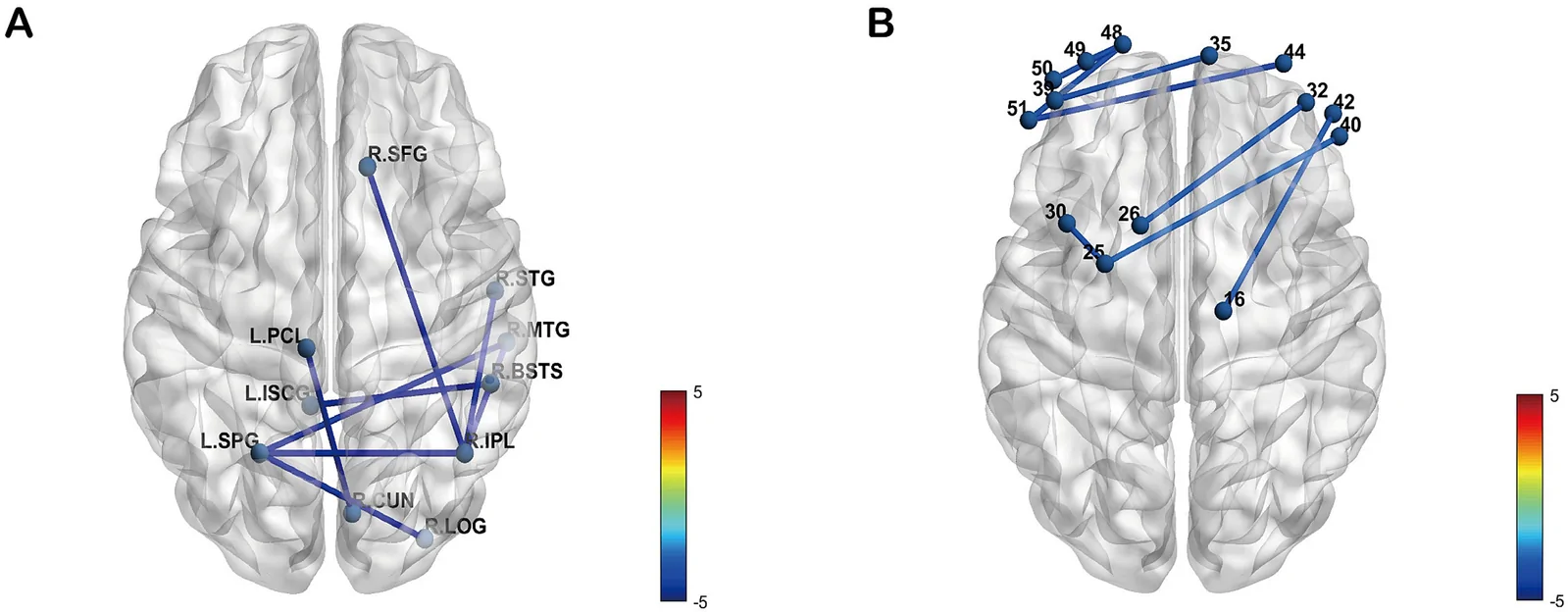
Functional connectivity difference maps between the EUD and HCs groups in EEG and fNIRS. (A) Delta-band FC (|*t*| > 4; 8 connections in total). (B) fNIRS FC (|*t*| > 3; 9 connections in total). The depicted connections indicate lower functional connectivity in the EUD group relative to HCs. EEG, electroencephalography; fNIRS, functional near-infrared spectroscopy; FC, functional connectivity.

### fNIRS functional connectivity

Channel-level fNIRS FC analysis was performed using 51 × 51 HbO2 connectivity matrices. Network-based statistic analysis identified a significant abnormal subnetwork in individuals with EUD compared with HCs, with a component-level NBS-corrected *P* value of .0409918. This subnetwork included 36 altered connections (*t* = −3.43 to −2.63, NBS_corrected *P* = .0409918, see Supplementary Table S2), all of which showed lower FC in the EUD group. The altered connections were primarily observed in frontal regions and were mainly characterized by cross-regional decoupling between functionally distinct frontal subregions. Cross-regional connections accounted for 91.7% of the abnormal fNIRS connections. Nine connections showed relatively strong effects (|*t*| > 3; see Figure 2B). The core affected systems included the ventrolateral prefrontal cortex (triangular/orbital regions of the inferior frontal gyrus), the dorsolateral prefrontal cortex, and the premotor and supplementary motor cortices; the connection with the lowest strength was observed between the orbitofrontal cortex and the orbital portion of the inferior frontal gyrus (ch44-ch51, *t* = −3.43, *P* < .001), with widespread reductions in connectivity both within these regions and between them and the premotor cortex and orbitofrontal cortex.

### Machine learning

Five machine learning models were constructed to discriminate patients with EUD from HCs, and the detailed classification metrics of all models are summarized in Table 2 and Figure 3. In this particular split of the data, the random forest model produced the numerically highest test-set AUC (0.80) among the 5 algorithms, with an accuracy of 0.67, precision of 0.75, recall of 0.67, and an F1 score of 0.71. Because the 5 models were compared on a single small held-out test set and no formal statistical test of AUC differences was performed, this result should be interpreted descriptively. In addition, the final model was selected on the basis of the highest cross-validated accuracy across 1000 random seeds, so the reported test-set metrics are likely to carry selection optimism and should be treated as exploratory.

**Table 2:** Comparison of classification metrics for different models.

**Models**	**AUC**	**Accuracy**	**Precision**	**Recall**	**F1_score**
**Decision tree**	0.77	0.73	0.81	0.72	0.76
**Random forest**	0.80	0.67	0.75	0.67	0.71
**XGBoost**	0.75	0.67	0.75	0.67	0.71
**LightGBM**	0.78	0.67	0.72	0.72	0.72
**SVM**	0.79	0.70	0.85	0.61	0.71

Abbreviation: SVM, support vector machine.

**Figure 3:**
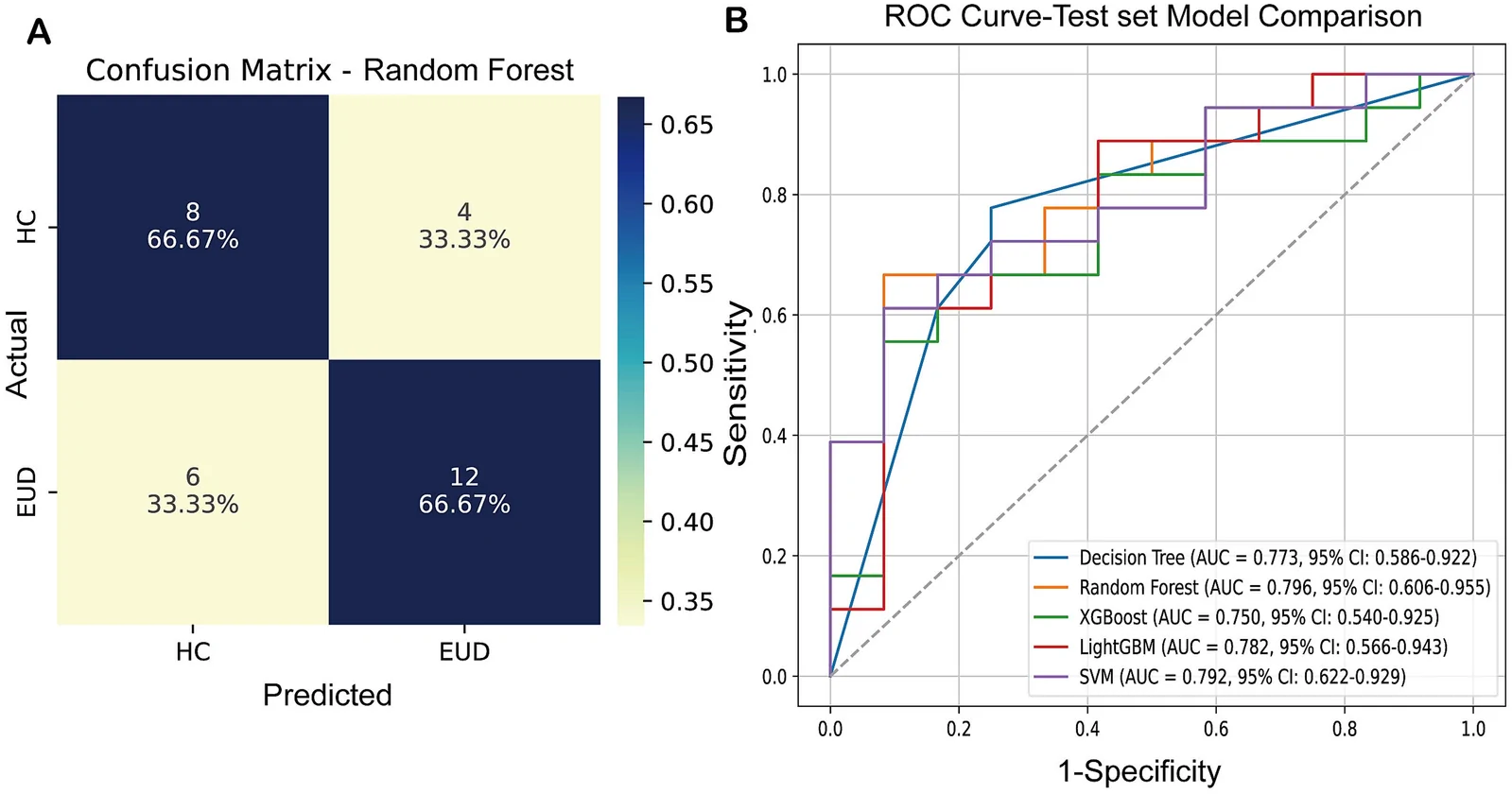
(A) Confusion matrix of random forest. (B) ROC curves for the test set.

### Correlation between functional connectivity and neuropsychological profiles

Within the EUD group, correlations were restricted to NBS-significant connections, with FDR correction applied within 4 prespecified families. The delta-band EEG substance-related family included withdrawal duration and the 3 DDQ subscales, yielding 21 × 4 = 84 tests. The delta-band EEG behavioral family included the total BIS-11 score and its 3 subscales, as well as the total BPAQ score and its 5 subscales, yielding 21 × 10 = 210 tests. The corresponding fNIRS families comprised 36 × 4 = 144 and 36 × 10 = 360 tests, respectively. The complete adjusted results are provided in Supplementary Table S3 and S4. After FDR correction, one association remained significant. Functional near-infrared spectroscopy connectivity between the primary motor cortex and the triangular part of the inferior frontal gyrus was negatively correlated with negative reinforcement craving (Spearman’s rho = −0.471, *P* = .0003; *p*_FDR = 0.047; 95% CI [−0.66, −0.23], see Figure 4). Several additional associations were observed at an uncorrected threshold. Twelve prefrontal-motor fNIRS connections showed negative correlations with negative reinforcement craving (rho = −0.418 to −0.269, *P* < .05). Two premotor-dorsolateral prefrontal connections were negatively correlated with drug craving intensity (rho = −0.327, −0.317, *P* < .05). In the EEG delta band, connectivity between the right middle temporal gyrus and the left supramarginal gyrus was positively correlated with withdrawal duration (rho = 0.411, *P* = .002). In addition, fNIRS connectivity between the orbitofrontal cortex and dorsolateral prefrontal cortex was negatively correlated with hostility, anger, cognitive impulsivity, and total BPAQ scores (rho = −0.292 to −0.285, *P* < .05, see Supplementary Table S3).

**Figure 4:**
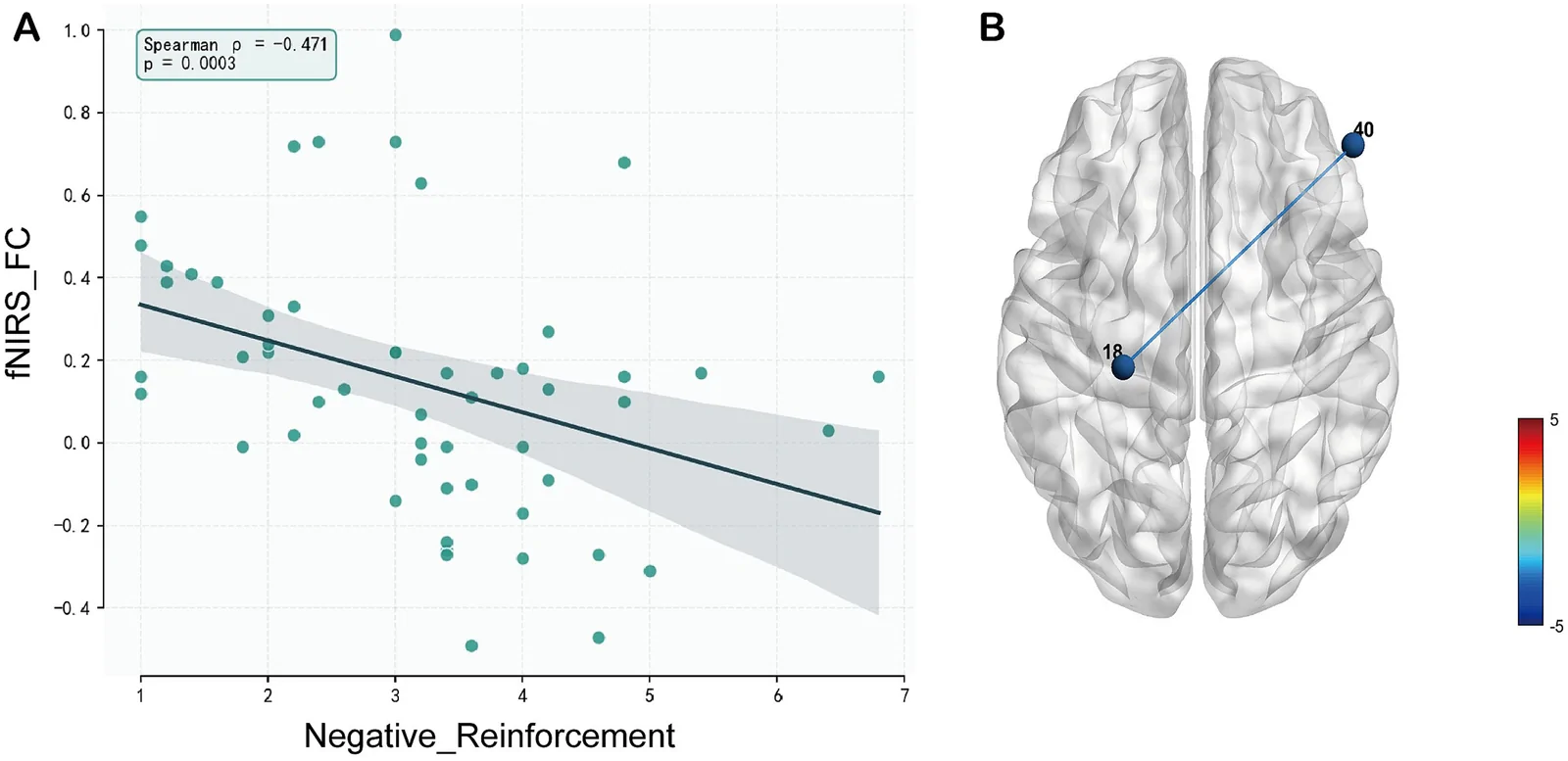
Functional connectivity between the triangular part of the inferior frontal gyrus (node 40) and primary motor cortex (node 18) negatively correlated with negative reinforcement scores. (A) Spearman correlation scatter plot. (B) Brain map showing the connectivity. (Spearman’s ρ = −0.471, *P* = .0003, *p*_FDR = 0.047).

Except for the association between primary motor cortex-inferior frontal gyrus connectivity and negative reinforcement craving, these correlations did not survive FDR correction. They are therefore reported as exploratory trends requiring validation in larger samples (see Supplementary Table S4).

## Discussion

This study combined resting-state EEG and fNIRS multimodal imaging techniques to investigate abnormalities in brain FC in male individuals with EUD and their association with clinical phenotypes. Three main findings emerged. First, individuals with EUD showed a distinct neuropsychological profile marked by higher cognitive and non-planning impulsivity, greater aggression, and stronger self-directed aggression. Second, source-level EEG analysis revealed delta-band-specific reductions in large-scale FC, primarily involving cross-network decoupling among default mode and dorsal attention. Third, fNIRS analysis showed widespread attenuation of frontal hemodynamic connectivity, particularly across prefrontal and motor-related subregions. Among the clinical associations, reduced prefrontal-motor connectivity was significantly related to stronger negative reinforcement craving after correction for multiple comparisons. Exploratory machine learning analyses further suggested that multimodal connectivity features contained information relevant to distinguishing EUD from HCs. Together, these findings support the view that EUD is associated with impaired functional integration across large-scale networks.

### Neuropsychological features

The neuropsychological findings indicate that EUD is associated with a selective pattern of behavioral dysregulation rather than a uniform increase across all impulsivity and aggression domains. Individuals with EUD showed higher total aggression, physical aggression, anger and self-directed aggression, whereas verbal aggression and hostility did not differ significantly between groups. This profile suggests that aggression in EUD is characterized by the co-occurrence of internalized (self-directed) and externalized (physical) manifestations. Substance use disorderss are highly comorbid with disorders such as impulsivity, aggression, and antisocial behavior.^11^ Previous research has indicated that internet addiction,^45^ alcohol misuse,^46^ and borderline personality disorder^47^ all exhibit a characteristic pattern of co-occurring internalized and externalized aggression. Furthermore, aggression shares a common neural substrate involving the prefrontal-limbic system.^13^ Externalized psychopathology is a strong predictor of early substance use.^48^ Regarding impulsivity traits, the EUD group exhibited significantly higher levels of cognitive impulsivity and non-planning impulsivity relative to HCs, whereas motor impulsivity was significantly lower. Higher cognitive and non-planning impulsivity reflect impaired cognitive control and foresight, which may predispose individuals to addictive behaviors and compromise their ability to sustain abstinence.^49^ The lower motor impulsivity in the EUD group may be related to the central inhibitory and sedative properties of etomidate, or behavioral withdrawal manifestations during the early abstinence phase. The direction of the motor impulsivity finding differs from that reported by Tang et al.^50^; This discrepancy may reflect differences between the independent cohorts and assessment periods. The present cohort was recruited and assessed in 2023, whereas the cohort in Tang et al. was collected during 2024-2025. There was no participant overlap between the 2 studies. These observations should be interpreted cautiously because the behavioral comparisons were exploratory.

### Delta-band network decoupling in EUD

The EEG findings point to delta-band network decoupling as the main electrophysiological alteration observed in EUD. Significant group differences were detected only in the delta band, whereas theta, alpha, beta, and low-gamma connectivity did not differ between groups. This frequency specificity is notable because delta-band activity is increasingly recognized as contributing to long-range coordination and temporal integration in large-scale brain networks, beyond its traditional association with sleep or anesthesia.^51^^,^^52^ Given that etomidate acts primarily through GABA_A receptor modulation, previous clinical anesthesia studies have found that etomidate anesthesia can induce EEG burst suppression and alterations in delta power spectral density.^53^^,^^54^ Altered delta-band connectivity may be relevant to the neurophysiological consequences of repeated exposure to a GABAergic anesthetic agent. However, because the present study was cross-sectional and conducted during early withdrawal, this interpretation should be considered hypothesis-generating rather than causal.

Previous simultaneous EEG-fNIRS and fMRI studies have revealed brain functional and topological abnormalities involving the default mode network, attention network, and sensorimotor network in EUD.^22^^,^^50^ The core pathology of addiction is not functional abnormality of a single brain region, but dysregulated coordination across multiple large-scale networks underlying reward, cognitive control, and attention.^55^^,^^56^ Under physiological conditions, the default mode network mediates internal self-referential processing, whereas the dorsal attention network supports externally directed attentional allocation for goal-directed behavior. The 2 networks maintain an antagonistic balance to enable flexible cognitive switching.^57–59^ The widespread cross-network decoupling between these 2 core networks observed in this study suggests impairment of this dynamic balance, which may be associated with addiction-related attentional bias and cognitive rigidity. Meanwhile, reduced connectivity between the hub nodes and visual/sensorimotor networks reflects the spread of impairment to perceptual systems, potentially connected to multidimensional cognitive deficits in addiction. These inferences remain to be validated in longitudinal studies.

### Frontal hemodynamic characteristics in fNIRS

The fNIRS findings extend the EEG results by showing reduced hemodynamic connectivity within frontal cortical systems. All significant fNIRS connections were weaker in individuals with EUD than in HCs, and most involved coupling between distinct frontal subregions. This pattern suggests that EUD is associated with reduced intra-frontal functional integration rather than isolated impairment of a single prefrontal area. The affected connections involved the dorsolateral prefrontal cortex, ventrolateral prefrontal cortex, orbitofrontal cortex, premotor cortex, and supplementary motor regions. These regions contribute to executive control, response inhibition, reward valuation, and action preparation, processes that are commonly disrupted in addictive disorders.^60^^,^^61^ Therefore, reduced coordination among these frontal lobe systems may partially explain the manifestation of high impulsive-aggressive behaviors in EUD. Further basic experiments and clinical longitudinal studies are required for verification.

The EEG and fNIRS findings provide convergent, but not interchangeable, evidence for impaired functional integration in EUD. Source-level EEG identified frequency-specific reductions in delta-band connectivity across distributed cortical networks, whereas fNIRS identified reduced hemodynamic connectivity within frontal cortical systems. These modalities therefore captured abnormalities at different spatial and physiological levels: EEG was sensitive to large-scale oscillatory coordination, while fNIRS provided a focused measure of prefrontal hemodynamic coupling.

### Clinical relevance and preliminary translational implications

The strongest clinical association in this study was the negative relationship between prefrontal-motor fNIRS connectivity and negative reinforcement craving. Specifically, weaker connectivity between the primary motor cortex and the triangular part of the inferior frontal gyrus was associated with stronger craving related to relief from negative affect or withdrawal-related discomfort. This finding is consistent with addiction models in which negative reinforcement contributes to persistent substance use.^62^^,^^63^ It also suggests that reduced coordination between inhibitory control and motor-related systems may be relevant to craving in EUD. However, this association should not be interpreted as evidence that the connectivity abnormality causes craving, because the present design was cross-sectional.

The machine learning results provide preliminary evidence that multimodal FC features carry discriminative information for EUD. The random forest model produced the numerically highest test-set AUC among the tested models in this particular split, but classification accuracy was moderate and no external validation cohort was available. These findings therefore support the potential value of EEG-fNIRS connectivity features for future objective assessment, but they do not establish a clinically deployable diagnostic biomarker. Larger independent cohorts, longitudinal designs, and intervention studies will be required to determine whether these connectivity measures can track symptom change, withdrawal recovery or treatment response.

In addition, under the uncorrected threshold, the study also suggests that brain networks may show a tendency to recover alongside the withdrawal process, as well as potential links between frontal-lobe regulation and aggressive phenotypes. These exploratory results mainly provide hypothetical directions for subsequent large-sample longitudinal studies.

### Limitations and future directions

Several limitations should be considered. First, this study recruited only male participants from a single center with a limited sample size, restricting the generalizability of the results. Future multicenter investigations incorporating female individuals and larger cohorts are required to validate our observations. Second, all individuals with EUD were assessed during early withdrawal, 3-14 days after their last etomidate use. Therefore, the observed neuropsychological and connectivity abnormalities may reflect a combination of trait vulnerability, repeated etomidate exposure, and withdrawal-related state effects. The cross-sectional design prevents these possibilities from being distinguished. Third, although simultaneous EEG-fNIRS provides complementary electrophysiological and hemodynamic information, both modalities have limited sensitivity to subcortical structures that are central to addiction, including the striatum and amygdala. Future studies integrating EEG-fNIRS with structural and functional MRI may provide a more complete account of cortico-subcortical circuitry in EUD. Fourth, residual confounding may arise from unadjusted clinical variables (etomidate use duration, daily dose, comorbid anxiety/depression, nicotine exposure) and the absence of objective wakefulness monitoring during eyes-closed recordings. Finally, the machine learning analysis was exploratory and lacked external validation. Because the 5 models were compared on a single small held-out test set without formal statistical testing of AUC differences, and because the final model was selected according to the highest cross-validated accuracy across 1000 random seeds, the reported test-set performance may carry selection optimism and should be interpreted cautiously. Larger independent cohorts and longitudinal designs are needed before these measures can be considered clinically useful biomarkers.

## Conclusions

This study used simultaneous resting-state EEG-fNIRS to characterize multimodal FC alterations in individuals with EUD. The findings suggest that EUD is associated with impaired functional integration, reflected by delta-band large-scale network decoupling and reduced frontal hemodynamic connectivity. The association between weakened prefrontal-motor connectivity and negative reinforcement craving further links these network alterations to a clinically relevant addiction phenotype. These results provide preliminary multimodal evidence for the neuroimaging features of EUD.

## Supplementary Material

Supplementary_Material_pyag049

## Data Availability

The datasets used and/or analyzed during the current study are available from the corresponding author on reasonable request.
