# Supplementary material for: Multimodal evidence for large-scale network imbalance in etomidate use disorder
Source: Int J Neuropsychopharmacol. 2026 Oct 14;29(11):pyag049. doi: 10.1093/ijnp/pyag049 (PMC13750372; doi:10.1093/ijnp/pyag049)
Supplement: Supplementary file 1 [file supplementary_material_pyag049.zip › Supplementary_Material_pyag049/Supplementary_Results_Table0929_pyag049.docx]

Table S1 Results of EEG functional connectivity

| No. | Brain Region 1 | Network | Brain Region 2 | Network | t-value | p-value |
| --- | --- | --- | --- | --- | --- | --- |
| NBS_delta3 | bankssts R | SMN | isthmuscingulate L | DMN | -4.6824948 | 9.12E-06 |
| NBS_delta13 | inferiorparietal R | DMN | superiorparietal L | DAN | -4.535671631 | 1.63E-05 |
| NBS_delta5 | inferiorparietal R | DMN | middletemporal R | DMN | -4.374066485 | 3.05E-05 |
| NBS_delta15 | middletemporal R | DMN | superiorparietal L | DAN | -4.146983701 | 7.17E-05 |
| NBS_delta14 | lateraloccipital R | VN | superiorparietal L | DAN | -4.113465525 | 8.12E-05 |
| NBS_delta12 | inferiorparietal R | DMN | superiorfrontal R | DMN | -4.11088896 | 8.20E-05 |
| NBS_delta17 | inferiorparietal R | DMN | superiortemporal R | SMN | -4.083936241 | 9.05E-05 |
| NBS_delta7 | cuneus R | VN | paracentral L | SMN | -4.025790431 | 0.000111986 |
| NBS_delta18 | isthmuscingulate R | DMN | supramarginal R | VAN/SN | -3.935831601 | 0.000155072 |
| NBS_delta21 | superiorparietal L | DAN | transversetemporal R | SMN | -3.880373853 | 0.000189096 |
| NBS_delta16 | cuneus L | VN | superiorparietal R | DAN | -3.835986801 | 0.000221349 |
| NBS_delta1 | bankssts R | SMN | caudalmiddlefrontal L | FPN | -3.73774681 | 0.000312365 |
| NBS_delta8 | inferiorparietal R | DMN | paracentral L | SMN | -3.699982613 | 0.00035604 |
| NBS_delta6 | cuneus L | VN | paracentral L | SMN | -3.621937053 | 0.000465345 |
| NBS_delta19 | middletemporal L | DMN | supramarginal R | VAN/SN | -3.59381874 | 0.000512001 |
| NBS_delta20 | middletemporal R | DMN | supramarginal R | VAN/SN | -3.564622227 | 0.000565112 |
| NBS_delta11 | paracentral L | SMN | parahippocampal R | DMN | -3.552373511 | 0.00058891 |
| NBS_delta4 | bankssts R | SMN | middletemporal L | DMN | -3.537785671 | 0.000618488 |
| NBS_delta2 | cuneus L | VN | fusiform R | VN | -3.513451394 | 0.000670973 |
| NBS_delta10 | inferiortemporal R | LN | parahippocampal R | DMN | -3.473898193 | 0.000765347 |
| NBS_delta9 | cuneus L | VN | parahippocampal R | DMN | -3.45082874 | 0.000826029 |

DMN (Default Mode Network), SMN (Sensorimotor Network), DAN (Dorsal Attention Network), VN (Visual Network), VAN/SN (Ventral Attention/Salience Network), FPN (Frontoparietal Control Network), LN (Limbic Network)。	Delta_Neg_Comnet_P=0.00159968

Table S2 Results of fNIRS functional connectivity

| Connection | CH1 | CH1 Brain Region (Brodmann) | CH2 | CH2 Brain Region (Brodmann) | p | t |
| --- | --- | --- | --- | --- | --- | --- |
| FNIRS32 | 44 | BA11 - Orbitofrontal area | 51 | BA46/47 - Dorsolateral prefrontal cortex/Inferior prefrontal gyrus | 0.000875721 | -3.433097044 |
| FNIRS27 | 48 | BA11 - Orbitofrontal area | 50 | BA46/47 - Dorsolateral prefrontal cortex/Inferior prefrontal gyrus | 0.002050371 | -3.167511047 |
| FNIRS26 | 35 | BA11 - Orbitofrontal area | 39 | BA46 - Dorsolateral prefrontal cortex | 0.002095873 | -3.160461967 |
| FNIRS11 | 48 | BA10 - Frontopolar area | 49 | BA46 - Dorsolateral prefrontal cortex | 0.002245505 | -3.13824587 |
| FNIRS7 | 26 | BA6 - Pre-Motor and Supplementary Motor Cortex | 32 | BA46 - Dorsolateral prefrontal cortex | 0.002620627 | -3.088086506 |
| FNIRS2 | 25 | BA6 - Pre-Motor and Supplementary Motor Cortex | 30 | BA6/8/9 - Pre-Motor/Frontal eye fields/Dorsolateral prefrontal cortex | 0.002847917 | -3.06085018 |
| FNIRS35 | 48 | BA11 - Orbitofrontal area | 51 | BA46/47 - Dorsolateral prefrontal cortex/Inferior prefrontal gyrus | 0.00291091 | -3.053658621 |
| FNIRS22 | 16 | BA6 - Pre-Motor and Supplementary Motor Cortex | 42 | BA46 - Dorsolateral prefrontal cortex | 0.003109059 | -3.031941296 |
| FNIRS17 | 25 | BA6 - Pre-Motor and Supplementary Motor Cortex | 40 | BA45 - pars triangularis Broca's area | 0.00338772 | -3.003474114 |
| FNIRS36 | 49 | BA11 - Orbitofrontal area | 51 | BA46/47 - Dorsolateral prefrontal cortex/Inferior prefrontal gyrus | 0.003578907 | -2.985169867 |
| FNIRS33 | 45 | BA11 - Orbitofrontal area | 51 | BA46/47 - Dorsolateral prefrontal cortex/Inferior prefrontal gyrus | 0.00369292 | -2.974679705 |
| FNIRS12 | 16 | BA6 - Pre-Motor and Supplementary Motor Cortex | 40 | BA45 - pars triangularis Broca's area | 0.003812661 | -2.963979596 |
| FNIRS4 | 26 | BA6 - Pre-Motor and Supplementary Motor Cortex | 31 | BA8 - Includes Frontal eye fields | 0.00447643 | -2.909754508 |
| FNIRS30 | 35 | BA10 - Frontopolar area | 51 | BA46/47 - Dorsolateral prefrontal cortex/Inferior prefrontal gyrus | 0.00456382 | -2.903175655 |
| FNIRS3 | 15 | BA4 - Primary Motor Cortex | 31 | BA8 - Includes Frontal eye fields | 0.004829111 | -2.883890783 |
| FNIRS8 | 26 | BA6 - Pre-Motor and Supplementary Motor Cortex | 33 | BA46 - Dorsolateral prefrontal cortex | 0.005471678 | -2.84093578 |
| FNIRS1 | 12 | BA4 - Primary Motor Cortex | 15 | BA4 - Primary Motor Cortex | 0.005659551 | -2.829251003 |
| FNIRS19 | 24 | BA6 - Pre-Motor and Supplementary Motor Cortex | 41 | BA45 - pars triangularis Broca's area | 0.005664223 | -2.828964912 |
| FNIRS9 | 29 | BA8 - Includes Frontal eye fields | 33 | BA46 - Dorsolateral prefrontal cortex | 0.005689597 | -2.82741308 |
| FNIRS23 | 42 | BA46 - Dorsolateral prefrontal cortex | 50 | BA46/47 - Dorsolateral prefrontal cortex/Inferior prefrontal gyrus | 0.00599918 | -2.832966551 |
| FNIRS31 | 43 | BA46 - Dorsolateral prefrontal cortex | 51 | BA46/47 - Dorsolateral prefrontal cortex/Inferior prefrontal gyrus | 0.006065457 | -2.805172286 |
| FNIRS14 | 19 | BA6 - Pre-Motor and Supplementary Motor Cortex | 40 | BA45 - pars triangularis Broca's area | 0.006203237 | -2.797333499 |
| FNIRS34 | 47 | BA10 - Frontopolar area | 51 | BA46/47 - Dorsolateral prefrontal cortex/Inferior prefrontal gyrus | 0.006304622 | -2.79166344 |
| FNIRS28 | 16 | BA6 - Pre-Motor and Supplementary Motor Cortex | 51 | BA46/47 - Dorsolateral prefrontal cortex/Inferior prefrontal gyrus | 0.006458358 | -2.783229872 |
| FNIRS16 | 24 | BA6 - Pre-Motor and Supplementary Motor Cortex | 40 | BA45 - pars triangularis Broca's area | 0.006954878 | -2.757181971 |
| FNIRS18 | 16 | BA6 - Pre-Motor and Supplementary Motor Cortex | 41 | BA45 - pars triangularis Broca's area | 0.006986185 | -2.755597043 |
| FNIRS6 | 21 | BA6 - Pre-Motor and Supplementary Motor Cortex | 32 | BA46 - Dorsolateral prefrontal cortex | 0.007662153 | -2.72286479 |
| FNIRS29 | 24 | BA6 - Pre-Motor and Supplementary Motor Cortex | 51 | BA46/47 - Dorsolateral prefrontal cortex/Inferior prefrontal gyrus | 0.007943189 | -2.710024749 |
| FNIRS5 | 28 | BA8 - Includes Frontal eye fields | 31 | BA8 - Includes Frontal eye fields | 0.008901236 | -2.669155271 |
| FNIRS15 | 24 | BA6 - Pre-Motor and Supplementary Motor Cortex | 40 | BA45 - pars triangularis Broca's area | 0.008984977 | -2.665775701 |
| FNIRS13 | 18 | BA4 - Primary Motor Cortex | 40 | BA45 - pars triangularis Broca's area | 0.009229904 | -2.65605151 |
| FNIRS20 | 29 | BA8 - Includes Frontal eye fields | 41 | BA45 - pars triangularis Broca's area | 0.009314152 | -2.652760746 |
| FNIRS10 | 21 | BA6 - Pre-Motor and Supplementary Motor Cortex | 39 | BA46 - Dorsolateral prefrontal cortex | 0.009323086 | -2.652413372 |
| FNIRS25 | 25 | BA6 - Pre-Motor and Supplementary Motor Cortex | 42 | BA46 - Dorsolateral prefrontal cortex | 0.009536361 | -2.651121991 |
| FNIRS21 | 30 | BA6/8/9 - Pre-Motor/Frontal eye fields/Dorsolateral prefrontal cortex | 41 | BA45 - pars triangularis Broca's area | 0.009773266 | -2.635287768 |
| FNIRS24 | 24 | BA6 - Pre-Motor and Supplementary Motor Cortex | 42 | BA46 - Dorsolateral prefrontal cortex | 0.009991023 | -2.627258678 |

Edge_Neg_Comnet_P=0.0409918

Table S3 Comparison of results before and after correction in the correlation analysis

| Variable name | Delta_FC | Spearman_rho | Spearman_p | Related fields | Significance level (uncorrected) | FDR_p value | Significance after FDR correction |
| --- | --- | --- | --- | --- | --- | --- | --- |
| Withdrawal_period | NBS_delta15 | 0.4106 | 0.002042 | Pos | p<0.01 | 0.16086 | Not significant |
| Drug_Control | NBS_delta6 | 0.3871 | 0.00383 | Pos | p<0.01 | 0.16086 | Not significant |
| Withdrawal_period | NBS_delta7 | -0.3386 | 0.012269 | Neg | p<0.05 | 0.343532 | Not significant |
| Withdrawal_period | NBS_delta9 | -0.3213 | 0.017853 | Neg | p<0.05 | 0.361654 | Not significant |
| Negative_Reinforcement | NBS_delta1 | 0.3122 | 0.021527 | Pos | p<0.05 | 0.361654 | Not significant |
| BPAQ2 | NBS_delta12 | 0.3774 | 0.004903 | Pos | p<0.01 | 0.582505 | Not significant |
| BIS1 | NBS_delta19 | 0.3331 | 0.01385 | Pos | p<0.05 | 0.582505 | Not significant |
| BPAQ | NBS_delta12 | 0.3313 | 0.014414 | Pos | p<0.05 | 0.582505 | Not significant |
| BIS3 | NBS_delta12 | -0.3297 | 0.014917 | Neg | p<0.05 | 0.582505 | Not significant |
| BPAQ5 | NBS_delta12 | 0.3261 | 0.016117 | Pos | p<0.05 | 0.582505 | Not significant |
| BPAQ1 | NBS_delta14 | -0.3246 | 0.016643 | Neg | p<0.05 | 0.582505 | Not significant |
| BPAQ1 | NBS_delta12 | 0.3121 | 0.021588 | Pos | p<0.05 | 0.64764 | Not significant |
| BPAQ2 | NBS_delta13 | -0.2893 | 0.033859 | Neg | p<0.05 | 0.797055 | Not significant |
| BIS3 | NBS_delta19 | -0.2886 | 0.034303 | Neg | p<0.05 | 0.797055 | Not significant |
| BPAQ1 | NBS_delta11 | 0.275 | 0.044147 | Pos | p<0.05 | 0.797055 | Not significant |
| BPAQ1 | NBS_delta6 | 0.2728 | 0.04595 | Pos | p<0.05 | 0.797055 | Not significant |
| BIS | NBS_delta2 | 0.2683 | 0.049834 | Pos | p<0.05 | 0.797055 | Not significant |
| Negative_Reinforcement | FNIRS13 | -0.4711 | 0.000324 | Neg | p<0.001 | 0.046656 | significant |
| Negative_Reinforcement | FNIRS16 | -0.4181 | 0.001653 | Neg | p<0.01 | 0.086832 | Not significant |
| Negative_Reinforcement | FNIRS24 | -0.415 | 0.001809 | Neg | p<0.01 | 0.086832 | Not significant |
| Negative_Reinforcement | FNIRS19 | -0.3636 | 0.006881 | Neg | p<0.01 | 0.247716 | Not significant |
| Drug_Craving | FNIRS25 | -0.3272 | 0.01575 | Neg | p<0.05 | 0.317686 | Not significant |
| Negative_Reinforcement | FNIRS29 | -0.3236 | 0.016997 | Neg | p<0.05 | 0.317686 | Not significant |
| Negative_Reinforcement | FNIRS17 | -0.3177 | 0.019224 | Neg | p<0.05 | 0.317686 | Not significant |
| Drug_Craving | FNIRS2 | -0.3167 | 0.019655 | Neg | p<0.05 | 0.317686 | Not significant |
| Negative_Reinforcement | FNIRS21 | -0.3097 | 0.022666 | Neg | p<0.05 | 0.317686 | Not significant |
| Negative_Reinforcement | FNIRS25 | -0.3047 | 0.025089 | Neg | p<0.05 | 0.317686 | Not significant |
| Negative_Reinforcement | FNIRS30 | -0.3022 | 0.026359 | Neg | p<0.05 | 0.317686 | Not significant |
| Negative_Reinforcement | FNIRS18 | -0.3009 | 0.02705 | Neg | p<0.05 | 0.317686 | Not significant |
| Negative_Reinforcement | FNIRS14 | -0.2979 | 0.02868 | Neg | p<0.05 | 0.317686 | Not significant |
| Negative_Reinforcement | FNIRS20 | -0.2802 | 0.040151 | Neg | p<0.05 | 0.391505 | Not significant |
| Negative_Reinforcement | FNIRS22 | -0.2689 | 0.049309 | Neg | p<0.05 | 0.391505 | Not significant |
| Hostility | FNIRS35 | -0.2918 | 0.032264 | Neg | p<0.05 | 0.997541 | Not significant |
| Anger | FNIRS35 | -0.2865 | 0.035672 | Neg | p<0.05 | 0.997541 | Not significant |
| Cognitive impulsivity | FNIRS35 | -0.2861 | 0.035967 | Neg | p<0.05 | 0.997541 | Not significant |
| BPAQ | FNIRS35 | -0.2845 | 0.037097 | Neg | p<0.05 | 0.997541 | Not significant |
| Verbal aggression | FNIRS10 | 0.2741 | 0.04493 | Pos | p<0.05 | 0.997541 | Not significant |

Table S4 Summary of Correlation Analysis Results

| Analysis | Log-transformed comparison | Unadjusted p<0.05 | FDR-significant | Strongest correlation |
| --- | --- | --- | --- | --- |
| BIS/BPAQ × Delta | 210 | 12 | 0 | Verbal aggression-delta12:ρ=0.377 |
| BIS/BPAQ × fNIRS | 360 | 5 | 0 | Hostility-FNIRS35:ρ=−0.292 |
| DDQ × Delta | 84 | 5 | 0 | Withdrawal Period-delta15:ρ=0.411 |
| DDQ × fNIRS | 144 | 15 | 1 | Negative reinforcement-FNIRS13:ρ=−0.471 |
